# Combining physical therapy and cognitive behavioral therapy techniques to improve balance confidence and community participation in people with unilateral transtibial amputation who use lower limb prostheses: a study protocol for a randomized sham-control clinical trial

**DOI:** 10.1186/s13063-019-3929-8

**Published:** 2019-12-30

**Authors:** McKenzie O. Bourque, Kristin L. Schneider, John E. Calamari, Christopher Reddin, Aaron Stachowiak, Matthew J. Major, Chad Duncan, Ranjini Muthukrishnan, Noah J. Rosenblatt

**Affiliations:** 1 Department of Psychology, Rosalind Franklin University of Medicine and Science, 3333 Green Bay Road, North Chicago, IL 60064 USA; 2 Captain James A Lovell Federal Health Care Center, 2450 Buckley Rd, North Chicago, IL 60064 USA; 3 Department of Physical Medicine and Rehabilitation, Northwestern University, Prosthetics and Orthotics Center, 680 North Lake Shore Drive, Chicago, IL 60611 USA; 4 Jesse Brown VA Medical Center, 820 S Damen Ave, Chicago, IL 60612 USA; 5 Edward Hines, Jr. VA Hospital, 5000 5th Ave, Hines, IL 60141 USA; 6 Dr. William M. Scholl College of Podiatric Medicines’ Center for Lower Extremity Ambulatory Research (CLEAR), Rosalind Franklin University of Medicine and Science, 3333 Green Bay Road, North Chicago, IL 60064 USA

**Keywords:** Amputee, Community integration, Activity, Fear avoidance, Virtual reality, Gaming

## Abstract

**Background:**

Low balance confidence is a prevalent yet overlooked issue among people who use lower limb prostheses (LLP) that can diminish community integration and quality of life. There is a critical need to develop rehabilitation programs that specifically target balance confidence in people who use LLP. Previous research has shown that multicomponent interventions including cognitive-behavioral therapy (CBT) techniques and exercise are feasible and effective for improving balance confidence in older adults.

Therefore, a cognitive behavioral–physical therapy (CBPT) intervention was developed to target balance confidence and increase community integration in people who use LLP.

**Methods/design:**

This randomized control trial will recruit 60 people who use LLP with low balance confidence. Participants will be randomized to the CBPT intervention condition or control condition.

**Discussion:**

The trial is designed to test the effects of the CBPT intervention on balance confidence and functional mobility in lower limb prosthesis users by examining self-reported and objective measures of community integration and quality of life. The trial will also examine the relationship between changes in balance confidence and changes in community integration following participation in CBPT intervention. Additionally, through participant feedback, researchers will identify opportunities to improve intervention efficacy.

**Trial registration:**

ClinicalTrials.gov, NCT03411148. Registration date: January 26, 2018.

## Background

Low balance confidence, defined as low self-perception in ones’ ability to maintain balance while performing specific activities, is a prevalent, yet overlooked issue among people who use lower limb prostheses (LLP). In a cohort of 435 community-dwelling individuals with lower limb amputation who had been living with LLP for at least 6 months, 65% reported levels of balance confidence below the threshold at which intervention is advocated for able-bodied adults [1, 2]. Low balance confidence can negatively impact community participation. In a cross-sectional study, low balance confidence was a significant predictor of social participation, even after accounting for mobility capability and other prosthesis-related characteristics [3]. Moreover, balance confidence at discharge from rehabilitation following a first amputation is a predictor of social participation 3 months after discharge [4], with low social participation significantly limiting quality of life [5, 6]. Low balance confidence is also associated with lower levels of physical activity [7].

Among people who use LLP, low balance confidence may be, in part, independent of functional ability; balance confidence and performance-based measures of balance are only moderately correlated [8] and, following discharge from rehabilitation, persons with a lower limb prosthesis continue to improve walking ability, while balance confidence does not continue to improve [4], suggesting the two may be independent. Accordingly, rehabilitation efforts that only target function may not necessarily improve balance confidence or increase community integration. In a 2014 study comparing two treadmill-based gait-training protocols for people who use LLP, participants demonstrated improved walking ability one month post-intervention (with no effect of intervention type) but reported no improvement in balance confidence [9]. Interventions that aim to improve community integration by alleviating restrictions due to low balance confidence may require multicomponent interventions that simultaneously address both physical ability and balance confidence.

A systematic review of 46 randomized control trials (RCTs) [10] and a separate meta-analysis of 24 RCTs [11] to improve balance confidence concluded that implementation of multicomponent interventions is feasible and effective in intact populations, particularly for persons with poor balance confidence. In most multicomponent interventions, physical abilities were addressed using physical therapy to target balance, gait, or strength; efficacy training (e.g., mastery experiences) increased the effects of physical therapy [11]. A major advantage of supplementing physical therapy exercises with cognitive behavioral therapy (CBT) strategies is that avoidance of activities can be directly targeted while addressing balance confidence. Previous interventions that have supplemented exercises with CBT demonstrated improvements in self-efficacy and increased activity up to 6 months post-intervention [11–13]. Supplementing exercises that specifically address balance ability with CBT strategies to address balance confidence may produce similar improvements in activity and self-efficacy in people who use LLP.

There is a critical need to develop and implement rehabilitation programs that specifically target balance confidence in order to increase community integration for people who use LLP. There are no current interventions to address balance confidence in this population. Therefore, we have developed a combined cognitive behavior and physical therapy (CBPT) intervention in which a behavioral therapist works in conjunction with a physical therapist to address underlying cognitions and physical skills that promote low balance confidence. Of particular note, the physical therapist will implement exercise in the intervention through the use of virtual reality (VR) games in the C-Mill Balance Suite (Motek; Netherlands). The games are displayed to the player on a 65-inch high-definition television screen at the front of the C-Mill, which is a 4 × 1 m (length × width) instrumented treadmill. Virtual reality games, in contrast to more traditional modalities, are used for several reasons. VR games provide a means to standardize progression based on performance and the ability to target multiple domains in few tasks while including a cognitive component, they can be adjusted for difficulty depending on the participants’ skill level, they encourage repetition of tasks and provide direct feedback in the form of a score, all of which promote motor learning [14, 15]. VR games are at least as effective as traditional physical therapy at improving balance and balance confidence in intact populations [16–18] and, moreover, are fun and motivating [14, 19, 20].

### Specific aims

#### Aim 1 (primary analyses)

Compare the effects of the combined cognitive behavior and physical therapy (CBPT) intervention condition versus a sham control condition on balance confidence and performance-based measures of balance and functional mobility in lower limb prosthesis users.

- *Hypothesis 1a*: Participants receiving CBPT intervention will demonstrate improvements in balance confidence over time compared to participants in the control condition.
- *Hypothesis 1b*: Performance-based measures of balance will improve after CBPT intervention.
- *Hypothesis 1c*: Performance-based measures of functional ability will improve after CBPT.

#### Aim 2 (secondary analyses)

Compare the effects of CBPT intervention versus a sham control on self-reported and objective measures of community integration and on quality of life.

- *Hypothesis 2a*: Participants receiving CBPT intervention will self-report and objectively demonstrate increased community integration over time compared to participants in the control condition.
- *Hypothesis 2b*: Participants receiving CBPT intervention will demonstrate improvements in quality of life compared to participants in the control condition.

#### Aim 3 (secondary analyses)

Examine the relationship between changes in balance confidence and changes in community integration following participation in CBPT intervention.

- *Hypothesis 3*: For participants in the intervention condition, change in balance confidence from 0 to 25 weeks will be directly correlated with changes in community participation (0 to 25 weeks).

#### Aim 4 (exploratory analyses)

Identify opportunities to improve intervention efficacy. This exploratory aim involves key informant interviews to identify themes related to residual barriers to social activity and mobility, the CBPT strategies that were deemed most effective, and additional strategies to address social participation and mobility barriers (e.g., behavioral activation, different physical therapy strategies).

## Methods/design

### Study design

**Fig. 1:**
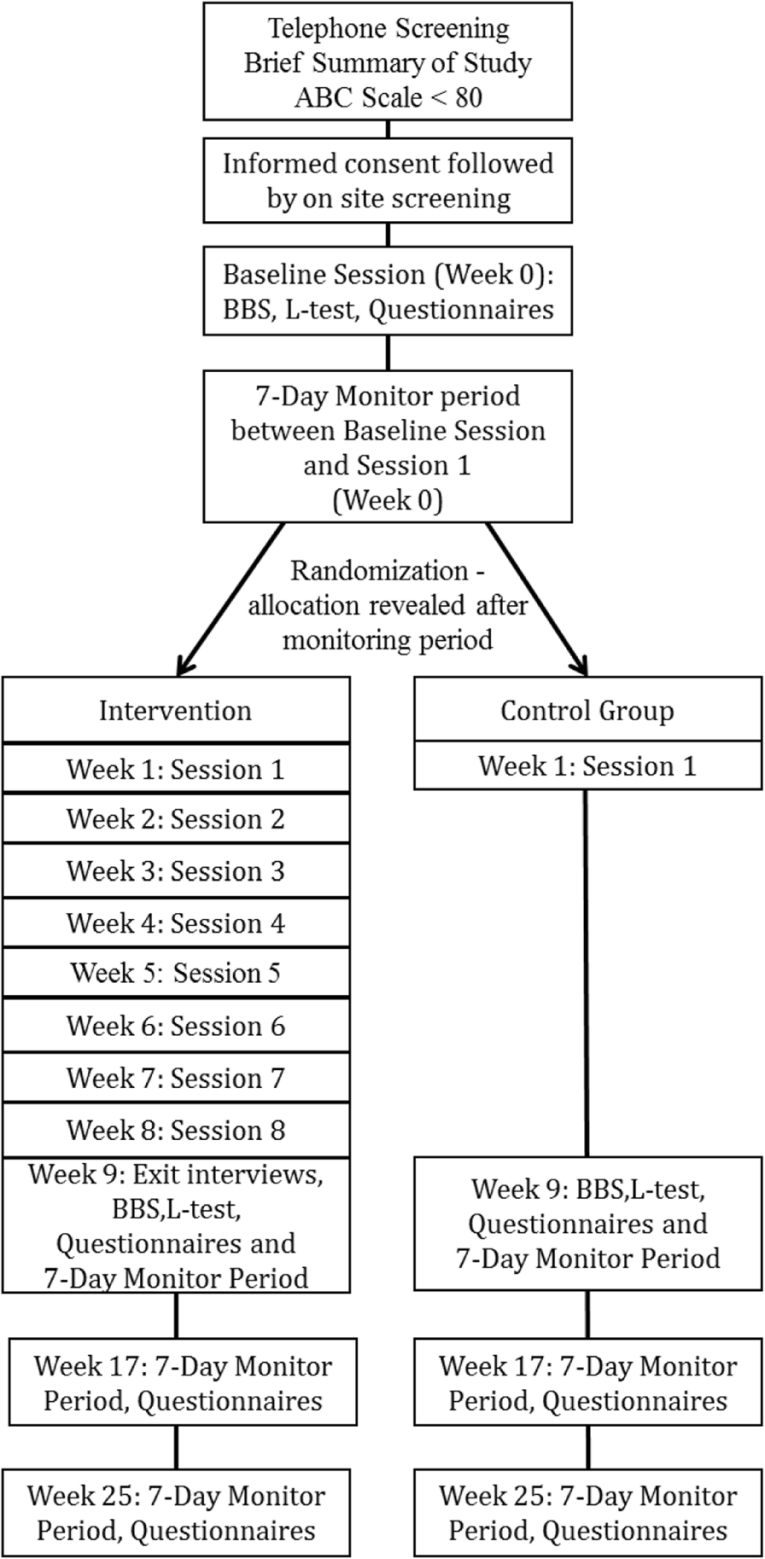
Flowchart of study participation

Sixty people who use LLP with low balance confidence will be randomized to the CBPT intervention condition or control condition (Fig. 1). Participants will be assigned to one of two randomization plans based on etymology of amputation: vascular causes (e.g., diabetes, peripheral vascular disease) or non-vascular causes (e.g., trauma, osteomylistis). For each plan, a block randomization will be used with a block size of 4 to ensure balance among the exercise groups throughout the study. The randomization plan will be developed by a researcher uninvolved with the project using www.randomization.com and sealed envelopes will be used to conceal the randomization schedule until enrolled participants are assigned to a group. For scheduling purposes, to ensure that the necessary clinicians are on site at the time that group assignment is revealed to the participant (at visit occurring ~ 1 week following enrollment), envelopes will be opened after the participant leaves the university after completing the enrollment visit.

Participants in the intervention condition will complete eight weekly training sessions that include a virtual reality active gaming component (i.e., PT component), with CBT strategies that increase balance confidence by addressing subject-specific avoidance behaviors and maladaptive cognitions. Each session will last approximately 1.5 h. Participants in the control condition will complete a series of at-home upper-body seated exercises during this 8-week period after being taught by a physical therapist how to perform the exercises. The intention of the sham control is to blind participants to the study group. At the start of the study and at 8 (post-intervention), 16, and 24 weeks following group allocation, participants will complete survey-based outcome measures and activity monitoring. At baseline and post-intervention, participants will complete performance-based assessments of balance and of functional mobility. Outcomes measures are described below.

To minimize loss to dropout between baseline and final follow-up, research coordinators will routinely contact participants by telephone and email to maintain engagement in order to maximize participant adherence. Adherence will be quantified based on number of study visits attended and frequency of attendance. If a participant wishes to drop out of the study, they will be queried as to their reasons for doing so and every attempt will be made to address their concerns and to obtain the minimum amount of data necessary to address hypotheses related to the primary outcome (i.e., self-reported measures at 0 and 25 weeks).

### Setting and ethics approval

The study will be performed at the Human Performance Laboratory at Rosalind Franklin University of Medicine and Science, North Chicago, IL, in conjunction with investigators at the Captain James A. Lovell Federal Health Care Center, North Chicago, IL, Edward Hines Jr. Veterans Administration Hospital, Hines, IL, and Northwestern University, Chicago, IL. The protocol described herein has already received institutional review board (IRB) approval from all study sites. Any modifications to the approved protocol or reporting of adverse events will be submitted to the IRB by the study PI according to published IRB policies.

### Participants

Participants will be veterans recruited directly from local VA Medical Centers or civilians/veterans recruited from the community. Recruitment at local VA hospital centers will take place through a two-step process. In step 1, medical records will be reviewed for persons having a unilateral transtibial amputation and prescribed prosthesis at least 6 months prior. Veterans found through medical record search will be sent a form letter indicating they may qualify for a study and should respond to the letter only if interested in additional information. The letter will ask them to complete and return a copy of the Activities-specific Balance Confidence (ABC) scale. In step 2, participants with ABC scores less than 80% will be called by research coordinators to determine eligibility through the use of an IRB-approved script. Recruitment of civilians/veterans from the community will primarily take place through flyers posted at local prosthetic clinics, support groups, and physical therapy offices.

All participants must meet the following criteria: at least 18 years of age, unilateral transtibial amputation, at least 6 months experience with using a definitive prosthesis, an ABC score less than 80. Participants will be excluded for open wounds on weight-bearing surfaces, inability to stand unassisted and weight-shift for 2.5 min (i.e., inability to perform the CBPT intervention), ill-fitting or ill-functioning prosthesis as determined during a screening with the study prosthetist, prohibited by primary care physician or research physician to engage in mild exercise, history of neurodegenerative diseases and stroke, or currently receiving physical therapy. No other concomitant therapies or interventions are excluded from the study and all other standards of care are permitted. Screening and all other study-related activities will occur only after the participant provides written informed consent, which will be taken by a study team member.

It is expected that 70 participants will be recruited in order to obtain a total of 60 eligible participants (30 intervention and 30 control participants), assuming a 15% drop-out rate. Using G*Power 3.1, a sensitivity analysis (with regard to Hypothesis 1a) was conducted, specifying a power of 0.80, an alpha of 0.05, and an average correlation of observations of 0.50. According to the analysis, with 30 participants in each group we will be able to detect a small to medium effect (f = 0.152) of intervention on ABC score over time with this design, which is consistent with the size of effect observed for older adults [10, 11]. We will have similar power for all hypotheses in Aim 2, which use the same analysis as Hypothesis 1a; we will have power to detect a medium effect size (d = 0.46) for Hypothesis 1b and a correlation of r ≥ 0.42 for Hypothesis 3.

### CBPT intervention

Below is a summary of the primary tasks undertaken during each of the eight sessions.

- *Session 1*: The initial goal of session 1 is to introduce the participant to the eight-session CBPT intervention and develop rapport. The behavioral therapist will deliver psychoeducation on how the use of the video games can improve balance as well as how balance confidence can impact behavior. Participants’ baseline physical skills on active video games will be assessed using two games (Trash Bin and Italian Alps; see Table 1 for a full description of the games) and the behavioral therapist will introduce the importance of goal setting in behavioral change. The behavioral therapist will introduce the *Behavior Recording Form for Prosthesis Use*, which is a standard behavior record with modifications to assess activity avoidance due to prosthesis concerns. Between-session assignment includes completion of the *Behavior Recording Form for Prosthesis Use* (Additional file 1)*.*
- *Session 2*: Session 2 will begin with a brief participant check-in regarding health and any falls during the prior week to ensure that the participant is safe to continue physical therapy. Next, the participant will be introduced to the four new video games not used during session 1 which will be played during the course of the intervention (Table 1). After completing the video games, the behavioral therapist will review the completed *Behavior Recording Form for Prosthesis Use* with the participant to gain an understanding of the participant’s activity restriction and avoidance behavior. Specific behavioral goals will be developed with the participant. The behavioral therapist will review the *Breathe to Relax* form with the participant, which teaches diaphragmatic breathing for relaxation (Additional file 2)

**Table 1:** Description of virtual reality games

Name of game	Description
Trash Bin Game	The player must shift their center of pressure (COP) left or right to move a trash bin to catch falling waste, which only falls vertically. The difficulty of the game can be increased by increasing the rate at which paper falls. The game can be played while shifting COP with feet in place, while side-stepping or while walking by adjusting spatiotemporal gait parameters. A screen capture is shown below
Traffic Jam	The goal of Traffic Jam is to allow cars, moving in two directions, to pass through an intersection, which is blocked when standing on both legs. The intersection clears by unloading one limb and shifting weight over the other limb; if cars are in the right lane then weight must be shifted to the left limb (*left yellow foot print* will disappear) and vice versa to clear the intersection. Traffic Jam focuses on limb loading and unloading and is only played while keeping both flat on the ground. A screen capture is shown below
Playing Soccer	Playing Soccer is initially played while shifting weight side to side and standing in place. Like the Trash Bin Game it can also be played shifting weight by side-stepping or by altering stepping parameters (step length, step width, and stance time) while walking. However, the side-stepping and walking options are not presented until later in the intervention. In all modes of the game, balls are dropped from the sky and fall downwards on the screen and the goal is to continuously bounce the ball off of a paddle, which can be moved by side-to-side shifting of the COP. The ball can ricochet off of side nets. For every ten bounces in a row an additional ball falls. Each gaming mode (standing, side-stepping, or walking) has three levels of difficulty defined by the length of the paddle (decreasing the length of the paddle increases difficulty). A screen capture is shown below
Playing Arkanoid	Playing Arkanoid is first played while standing in place. It can also be played in side-stepping mode or walking mode, but these modes are not shown until later in the intervention. In Playing Arkanoid, a ball is dropped from the sky and falls downwards on the screen. The goal is to continuously bounce the ball off of a paddle so that the ball hits and breaks a series of bricks at the top of the screen; after hitting the bricks the ball again falls down to the paddle. The paddle can be moved by shifting the COP. The ball can bounce off of side walls. A screen capture is shown below
Forest Walk	Forest Walk is played only in walking mode. A forest scene is projected on the treadmill surface and animals and other objects appear and travel with the speed of the treadmill belt; the goal is to make on-line adjustments to the gait pattern in order to avoid stepping on the animals or other objects while intentionally stepping on projected stars and soccer balls; points are awarded for stepping on the proper objects and points are subtracted for stepping on all other objects. The game targets gait adaptability. An overhead view of the treadmill is shown below
Italian Alps	The Italian Alps game is only played in walking mode. In Italian Alps, the player “pushes” a cart through a street and must collect ingredients to make a pizza (see bar across the bottom horizontal bar) to make a pizza while avoiding crashing into other objects such as flower boxes. The motion of the cart tracks the center of pressure (COP) and the flow of the scene is dictated by walking speed. A screen capture is shown below

Between-session assignments will include completion of the *Behavior Recording Form for Prosthesis Use*, reviewing the *Breathe to Relax* form, as well as practice the breathing skill.

- *Session 3*: After a brief check in to ensure the participant is well, the participant will play all four video games from Session 2. The behavioral therapist will then conduct an in-depth review of between-session assignments to continue skill building in behavior recording and goal setting. Additionally, the behavioral therapist will review and practice diaphragmatic breathing with the participant as well as refine initial behavioral goals. The behavioral therapist will then introduce systematic exposure to the participant in the context of reducing fear and changing behavior. This will be followed by an exposure exercise. The exposure exercise will be developed through a collaborative process between the behavioral therapist and the physical therapist based on an emerging understanding of the participant’s fear exposure hierarchy and physical ability. A fear exposure hierarchy is composed of a series of gradated tasks based on level of provoked fearfulness. An example of an exposure exercise is asking a participant that avoids reaching for an object above their head to practice reaching up high. Exposures will be conducted in the lab under the guidance of the behavioral therapist as well as at home in between sessions.

Between-session assignments will include completion of the *Behavior Recording Form for Prosthesis Use*, practicing *Breath to Relax*, and an at-home exposure exercise.

- *Sessions 4 through 7*: The physical therapist will continue to work on games with the participant. The behavioral therapist will continue to use and review previously introduced procedures, including physical skill acquisition, during physical therapy intervention, behavior recording, diaphragmatic breathing, and community-based exposure exercises.

Between-session assignments continue to include completion of the *Behavior Recording Form for Prosthesis Use*, using the *Breath to Relax* form, and performing an at-home exposure.

- *Session 8*: The final session will focus on summarizing the participant’s behavior change goals and progress made towards completing goals, as well as physical progress in the component and any learned behavioral strategies. The behavioral therapist will discuss strategies to continue to further gains as well as strategies for preventing relapse.

#### Key informant interview

At the end of the CBPT intervention (or at drop-out), participants who received the intervention will complete a 30–60 min, recorded, individual interview with investigators to discuss their experience with the intervention. A semi-structured interview will be used to assess the following areas.

#### Intervention experience

Participants will be asked to discuss what they liked and disliked about the intervention, their preference for the length of the intervention and the length of the individual sessions, the reasons they attended or missed sessions, their thoughts on combining the CBT and PT strategies, and the role of the CBT and gaming exercises in addressing their balance confidence and activity restriction. We will also obtain feedback on the CBT forms and assignments, including information on comprehension, burden, and usefulness for addressing balance confidence and activity restriction.

#### Barriers to participation

Participants will be asked about barriers to integration that they felt were not addressed during the intervention, as well as what the intervention could include to address those barriers.

#### Opinions on recruitment

Participants will be asked about advertising messages and recruitment venues that would increase the appeal of participating in similar future studies.

### Training and oversight of behavioral therapist and physical therapist

Two advanced, clinical psychology doctoral students will be trained by two licensed clinical psychologists to deliver the CBT portion of the intervention. The behavioral therapists will review the treatment manual, which will be followed by four 2-h training sessions covering balance confidence, CBT, the integration of CBT into a PT setting, and understanding the psychological and physical health sequelae of a leg amputation. Two training sessions will focus on the delivery of the CBT strategies. This will include a review of CBT theory and specific CBT strategies used to address balance confidence and activity restriction. In addition, behavioral therapists will role-play the delivery of the CBT strategies to ensure intervention fidelity and to provide feedback on adherence of the intervention protocol. Potential problems with the delivery of the CBT content will be discussed (e.g., participant fails to routinely complete assignments).

Once the intervention begins, behavioral therapists will receive supervision by licensed clinical psychologists. Supervisors will listen to audio recordings of the first eight sessions for each behavioral therapist and provide corrective feedback; thereafter, 10% of sessions will be reviewed. The behavioral therapists will also attend weekly supervision meetings to discuss their participant’s progress and ensure fidelity to the intervention. Behavioral therapist and auditor checklists for each session will be used to ensure that treatment objectives for each CBPT session are met. The behavioral therapist will complete the behavioral therapist checklist after each session. A random selection of audio-recorded sessions for each participant will be audited by supervisors to monitor treatment fidelity. They will complete the appropriate auditor checklist. When a session is reviewed with less than 85% of treatment-specific objectives met, supervisors will remediate training as needed. This process will continue through all treatment waves so that drift can be swiftly corrected.

The research teams’ senior licensed physical therapist will train a physical therapist to complete the PT components of the intervention. After reviewing the training manual, the sessions will include training on the operation system used for administering physical therapy exercises and all safety features in the laboratories. The physical therapist will also learn how to recognize adverse events, including excessive exertion, and be taught to monitor and assess vital signs throughout the intervention to avoid excessive exertion. Most importantly, the physical therapist will be instructed on how to regress and progress the level of difficulty for each game following the protocol.

### Sham-control condition

Participants assigned to the control condition will be introduced to three exercise modules (Modules A–C) that include seated exercises to improve core and arm strength and flexibility. All of the exercises are demonstrated to the participant by a physical therapist at the initial session. Participants will be provided a manual with thorough descriptions and visual aids of the seated exercises. Some of the exercises include use of a resistive (plastic air-filled) ball, which will also be provided to the participant. Participants will be instructed to start with Module A and continue for weeks 1–3, followed by Module B for weeks 4–6, and to finish with Module C for weeks 6–8. Participants will be told to follow the modules at their own pace. Each module includes approximately eight exercises that take 15–20 min to complete and participants will be told to work on the exercises three days per week. Participants will be provided a worksheet to record exercise days. Participants will be contacted by phone every 2–3 weeks during the 8-week intervention period to maintain engagement and encourage completion of the follow-up assessments.

The control is intended to account for a potential placebo effect and is considered a sham-control in that none of the exercises target lower extremity strength or flexibility, which is more likely to induce functional improvements and promote balance confidence during weight-bearing activities.

### Outcome measures

Outcomes will be collected before the start of the intervention (baseline) and again at 8 weeks (post-intervention), 16 weeks, and 24 weeks following group allocation.

#### Balance confidence (primary outcome measure)

Balance confidence will be measured through the Activities-specific Balance Confidence (ABC) scale. The scale asks participants to rate their level of confidence in completing 16 complex functional tasks without losing balance [21]. It has strong reliability (ICC = 0.91), internal consistency (Cronbach α = 0.95), and good construct validity in samples of older adults with fear of falling [22]. The ABC scale has also been validated to assess balance confidence in individuals with lower limb amputations [1].

#### Community integration

The following measures will be used to quantify community integration (secondary outcomes).

#### Activity monitoring

Participants will receive a SmartWatch3 Activity Monitor (SAM; Modus Health, WA, USA), which records stride count data for every minute of use, and a QStarz BT-Q1000XT (Qstarz International, Taipei) GPS travel recorder, which records a participant’s longitude, latitude, local time and date, and position every 10 s. The SAM and GPS will be worn for one week on the prosthetic pylon and then returned in a pre-paid envelope. Community integration will be assessed as the average number of steps/day, average number of steps/day taken outside the home, and total time spent outside the home per day. The individual who will link the GPS and activity monitor data to calculate steps/day as well as steps/day taken outside the home and total time spent outside the home per day will only be sent coded data so as to be blinded to the group inclusion.

#### Community Reintegration of Service members

Community Reintegration of Service members (CRIS) measures activity and participation with three subscales, extent of participation, perceived limitation, and participation satisfaction. The first two of the three scales are included in this study. The extent of participation scale evaluates the frequency of engaging in specific activities using seven-point scales (e.g., “In the past two weeks on average, how often did you engage in recreational activities, not including watching TV?”). The perceived limitation scale uses seven-point scales to evaluate self-perceived limitations in participation (e.g., “I avoided going to crowded places such as the mall of community gatherings”). The CRIS has been validated in a sample of injured veterans [23].

#### Short-Form Health Survey (SF36)

The SF36 consists of 36 questions that measure eight dimensions of health-related quality of life [24]. Three subscales, role limitation due to physical health problems, social functioning, and role limitations due to emotional problems, will be separately examined as secondary outcomes to assess community integration.

#### Frenchay Activity Index

The Frenchay Activity Index (FAI) is a 15-item behavioral scale that primarily measures social participation and daily activities (i.e., domestic/chores, work/leisure, and outdoor activities) [25]. Frequency of participation in activities over the last 3–6 months is rated on a four-point scale resulting in a single summary score, ranging from 0 (inactive) to 45 (highly active). The FAI is a valid and reliable measure among users of a LLP (ICC = 0.79) [26]. We will include the measure based on its association with the ABC scale in users with a LLP [4].

Additional secondary outcomes include quality of life and functional ability as described below.

#### Quality of life

The Well-being scale of the Prosthetic Evaluation Questionnaire (PEQ) will be used to assess quality of life (QoL) [27]. The PEQ is a survey that was developed to quantify function and prosthesis-related QoL for persons with lower limb amputation. The user marks a visual analog scale with two anchor points to respond to the following: “Over the past four weeks rate”…1) “How satisfied have you been with how things have worked out since your amputation” (anchors “extremely dissatisfied” and “extremely satisfied”)? and 2) “Your quality of life” (anchors “worst possible life” and “best possible life”). The simplified assessment allows participants to easily provide a summary of experiences by essentially considering QoL as unidimensional. This particular scale has good consistency (Cronbach α = 0.83) and reliability (ICC = 0.89) [28].

#### Functional abilities

The Berg Balance Scale (BBS) and L-test of walking will be used to measure functional abilities. Blinded assessors with prior training in administering these measures will assess performance.

- *Berg Balance Scale*: This test requires participants to complete 14 tasks of increasing difficulty beginning with sit-to-stand and progressing to unipedal stance. It is a well-established outcome measure initially designed to assess balance in older adults [29] and has been validated for persons with lower limb amputation [8]. A blinded assessor who is unfamiliar with the study protocol will perform the assessments at baseline and follow-up.
- *L-test*: Participants rise from a chair without armrests, walk 3 m, turn right, walk 7 m, turn around and trace their path back to the start. The time to complete the task is recorded. The test has strong reliability (ICC = 0.98) and convergent validity (r = 0.93 with the Timed-up-and-Go test (TUG) [30]. The L-test is better than TUG at distinguishing changes in walking ability of fit, younger LLP users and it is as strong a predictor of social participation as is balance confidence [4]. The same blinded assessor who conducts the Berg Balance Scale will conduct the L-test.

### Data management and sharing

Data collection methods include the use of: 1) commercially available step count monitors and GPS trackers; 2) completion of pen-and-paper versions of validated clinical surveys; 3) scores on validated performance-based functional tests of balance. The methods for managing each type of data are nearly identical. Specifically, when a potential participant arrives at a study site to obtain informed consent, they will be assigned a study ID number that contains no identifiable information. Should that participant enroll in the study, then all data will be labeled and referenced only with the study ID number. For example, data from the monitors will be downloaded onto password-protected computers, assigned the appropriate ID, and then analyzed offline. All pen-and-paper data will only have ID numbers listed on them and no identifiable information (e.g., participant name) to ensure confidentiality. A single list linking names and codes will be kept on a password-protected computer locked in the PIs laboratory. All performance based data and pen-and-paper based outcomes data will be entered into an IRB-approved secure on-line data collection tool, Qualtrics, using the appropriate subject ID. Summative scores from Qualtrics will then be entered into electronic files on password-protected computers to further ensure confidentiality. De-identified data on Qualtrics can be shared among all research team members.

The study PI will periodically check the data values to ensure they are within range and will verify entries based on the original pen-and-paper versions, which will be stored in locked cabinets in the PI’s laboratory to further ensure confidentiality. All data will be stored for no less than 3 years following submission of the final financial report. A separate data monitoring committee is not required by the IRB or funding source for this study. However, the Human Research Protection Office (HRPO) of the US Army Medical Research and Development Command can perform an on-site audit at any time. To ensure confidentiality, information about an enrolled participant will only be shared with team members unless instructed otherwise by the HRPO.

While the final dataset will be stripped of identifiers prior to release for sharing, there remains the possibility of deductive disclosure of subjects given the somewhat small number of persons with transtibial amputation in the Chicago-land VHA system meeting the inclusion criteria of the study. Thus, we will make the final research data (both the raw and processed data) and any relevant documentation available to users only under a data-sharing agreement requiring investigators to commit to: 1) using the data only for research purposes and not for purposes of identifying participants; 2) properly securing the data; 3) destroying or returning the data after analyses are completed; 4) an agreement to share any new data sets resulting from the initial sharing agreement. The research and clinical community will be made aware of the data when the study results are presented at scientific meetings and in final publication form.

### Study administration

This trial will not include a coordinating center nor a Trial Steering Committee and there is no Stakeholder and Public Involvement Group involved. The study PI will undertake all administrative oversight, aside from that provided by the local IRB. He will have regular meetings with all investigators to discuss problems as they arise as well as progress of the study. The study PI will work with a study coordinator to identify potential recruits and consent will take place by any of the listed investigators on the study.

### Analysis plan

To test Hypothesis 1a we plan to use a 2 (group) × 4 (time point) mixed ANOVA to examine if there are significant differences in changes in ABC scores between the groups (i.e., an interaction effect), accounting for any missing data. In the event of a significant interaction, a post-hoc paired *t*-test will be used to compare ABC scale in the intervention group at 0 and 25 weeks and independent samples *t*-test to compare ABC scale between groups at 25 weeks.

To test Hypotheses 1b and 1c we will use a paired *t*-test to compare BBS scores and L-test times pre- and post-intervention. H1b and c do not compare time-related changes in performance between groups as they are only intended to assess the immediate effects of intervention.

The same analyses from Hypothesis 1a will be used to test all hypotheses in Aim 2. For Hypothesis 2a, a group × time interaction on self-reported community participation measures (CRIS, FAI, SF36 scales) and objective measures from the activity monitors will be tested. A false-discovery rate correction method will be used to minimize the likelihood of type I errors. For Hypothesis 2b, an interaction on the QoL score will be tested. In the event that H2a is not supported, alternative measures to assess integration will be considered.

To test Hypothesis 3 changes in ABC scores (0 weeks to 25 weeks) will be correlated with changes in self-reported and objective measures of community participation. A false-discovery rate correction method will be administered to minimize the likelihood of type I errors. In the unlikely event that Hypotheses 1a and 2a are not supported, this would not exclude the possibility of an association between subject-specific changes in confidence and participation. In such a case, a significant correlation would suggest that improving balance confidence can increase participation, but for optimum effectiveness of the intervention, one should only train individuals who are most likely to respond to the intervention (show large increase in ABC scores). As an initial step to identify commonality among “responders” we would employ a suite of supervised learning techniques (e.g., cluster and canonical correlation analysis) to explore multidimensional relationships among responses to individual items on the ABC, BBS, CRIS, and SF36.

To analyze intervention efficacy, the interviews will first be transcribed verbatim. After 15 interviews, all investigators will review the transcripts to generate an initial list of codes. After five additional interviews, they will again re-view transcripts to assess whether new codes emerged. If saturation is not reached after 20 interviews, another five participants will be interviewed and saturation will be reassessed. Once saturation is reached, lead investigators will review all transcripts to generate an initial list of codes using a content coding framework. The creation of the coding framework will begin by the identification of anything that participant’s mention related to the CBT strategies, the gaming exercises, activity restriction, or fear of falling/balance confidence. From this broad coding framework, responses will be sorted according to the themes. Brief descriptions of final codes will be created. A lead investigator will then meet with research assistants to discuss the coding structure. Research assistants will code three transcripts, meet to review discrepancies, and revise the codes, if necessary. Remaining transcripts will be coded individually; research assistants will then meet to resolve discrepancies. Final codes will be entered into NVivo, version 9 for analysis.

## Discussion

This paper describes a randomized clinical trial comparing the effect of a novel CBPT intervention with that of a sham control condition on balance confidence and community integration for persons using LLPs. If successful, the proposed work has the potential to advance the field of prosthetic rehabilitation by integrating CBT and PT strategies into an intervention that will improve balance confidence, limit associated activity avoidance, and ultimately promote community integration. Integrating CBT and PT strategies within the rehabilitation of persons using LLPs is a fairly novel approach, but one that has demonstrated feasibility and effectiveness in the clinical setting among community-dwelling older adults, as well as elderly residents in assisted living centers [17, 31, 32]. Including a CBPT intervention early in rehabilitation may be critical as balance confidence is unlikely to change thereafter [4, 9].

Medical comorbidity is common in older individuals with LLP [33]. Issues related to complicated medical comorbidities may cause challenges in recruitment, such as scheduling difficulties due to unique physical health concerns. For example, participants with medical comorbidities may already have to manage many weekly medical appointments, which can pose a greater difficulty in scheduling intervention sessions. The research coordinator will work closely with participants to manage appointments and schedules. Additionally, the research team will have multiple trained behavioral therapists and physical therapists with varied availability to accommodate participant schedules. Unique physical concerns will be addressed by contacting participant’s providers to gain further medical clearance. If necessary, and with the participants permission, research team members (e.g., physical therapist, research prosthetist) will communicate directly with the participant’s providers. In order to accommodate participant schedules, it may be necessary to modify the number of intervention sessions. Although modifications may impact fidelity to the study protocol, including a representative sample with a variety of medical comorbidities will enhance generalizability.

The results of this trial will be submitted for publication in peer-reviewed journals and presented at local and national meetings regardless of outcome.

### Limitations

Several factors may limit the impact of the work. The results may not generalize to persons using LLPs due to transfemoral and/or bilateral amputation. We will exclude these groups to avoid between-subject variability due to differences in components and residual length, which are exacerbated in these excluded groups. However, based on prior work demonstrating no effect of level of amputation on ABC score, [23] if successful, a similar intervention could improve balance confidence in these groups as well. However, future work would be needed to test the efficacy of CBPT intervention with these groups. In addition, the use of a multicomponent intervention precludes us from quantifying the extent to which CBT or PT components alone act to improve balance confidence, or if (and how) the combined effects interact. A future disentanglement study would be needed to determine whether, for example, CBT or active video games alone provide similar results. In addition, future studies will also need to demonstrate clinical feasibility by utilizing standard of care PT rather than a sham control condition as a comparator (Additional file 3).

## Conclusions

The results of this study will provide information on the initial efficacy, feasibility, and impact of a combined CBT and PT intervention on balance confidence and community integration in adults with a LLP. If efficacious, increased support for novel care approaches in which behavioral therapists are integrated into other outpatient clinic settings will be necessary for further dissemination and implementation.

## Trial status

Current protocol is SCPM 17–023, version 2.3, which was last updated on June 3, 2019. Recruitment began on April 5, 2018 with an anticipated recruitment completion date of December 31, 2020.

## Supplementary information

**Additional file 1.** Behavior Recording Form for Prosthesis Use.

**Additional file 2.** Breath To Relax Description.

**Additional file 3.** SPIRIT 2013 Checklist: Recommended items to address in a clinical trial protocol and related documents.

## Data Availability

The protocol manual is available upon request.

## Abbreviations

ABC scale: Activities-specific Balance Confidence scale
BBS: Berg Balance Scale
CBPT: Cognitive behavioral–physical therapy
CBT: Cognitive–behavioral therapy
CRIS: Community Reintegration of Service members
FAI: Frenchay Activity Index
HRPO: Human Research Protection Office
IRB: Institutional review board
LLP: Lower limb prosthesis
PEQ: Prosthetic Evaluation Questionnaire (PEQ)
QoL: Quality of life
RCT: Randomized control trials
SF36: 36 question Short-Form Health Survey
TUG: Timed-up-and-Go test
VR: Virtual reality
