# Supplementary material for: Combining physical therapy and cognitive behavioral therapy techniques to improve balance confidence and community participation in people with unilateral transtibial amputation who use lower limb prostheses: a study protocol for a randomized sham-control clinical trial
Source: Trials. 2019 Dec 30;20:812. doi: 10.1186/s13063-019-3929-8 (PMC6937857; doi:10.1186/s13063-019-3929-8)
Supplement: Supplementary file 1 — Additional file 1. Behavior Recording Form for Prosthesis Use. [file 13063_2019_3929_MOESM1_ESM.docx]

***Additional file 1- Behavior Recording Form for Prosthesis Use – COMPLETE AFTER SESSION 1***

*The purpose of this form is to gather information about activities you might avoid or do less often because of concerns you have around the use of you prosthesis. The intent is to* ***make note of situations during the next week where you might have limited or avoided something because of prosthesis use concerns****. You are asked to indicate the date, time, and situation you were in when concerns about using your prosthesis came up. You are also asked to note what you were thinking at the time. By carefully tracking these situations, you may become more aware of how anxiety or nervousness you have about different situations impacts you.*

| *Date/Day of week* | *Time* | *What were you*  *doing?* | *What were your thought or concerns?* | *What behavior was affected?* | *How did you feel afterward?* |
| --- | --- | --- | --- | --- | --- |
|  |  |  |  |  |  |
|  |  |  |  |  |  |
|  |  |  |  |  |  |
|  |  |  |  |  |  |
|  |  |  |  |  |  |
|  |  |  |  |  |  |
|  |  |  |  |  |  |
|  |  |  |  |  |  |
