# Supplementary material for: Combining physical therapy and cognitive behavioral therapy techniques to improve balance confidence and community participation in people with unilateral transtibial amputation who use lower limb prostheses: a study protocol for a randomized sham-control clinical trial
Source: Trials. 2019 Dec 30;20:812. doi: 10.1186/s13063-019-3929-8 (PMC6937857; doi:10.1186/s13063-019-3929-8)
Supplement: Supplementary file 2 — Additional file 2. Breath To Relax Description. [file 13063_2019_3929_MOESM2_ESM.docx]

**Additional file 2 - Breathe to Relax**

*Overview:*

When people become nervous, anxious, or distressed it affects how they think and act. When these reactions go unchecked, they can result in even more anxiety. The purpose of the *Breathe to Relax* is to counter this reaction so that you feel less distressed and can continue to pursue the activities of life you choose to do. While *Breathe to Relax* will not to make all anxiety go away, it will allow you to manage it more effectively.

The *Breathe to Relax* procedure involves two skills:

1) Learning to notice your breathing and how to slow it down by breathing deeply into your lungs

2) Learning to remove your focus from negative thoughts that cause distress and focusing your attention

in the present moment.

*At home practice exercises:*

You should practice relaxation skills in a quiet place in your home for approximately10 minutes each day

**Step 1:**

Sit in a comfortable chair. Place one hand on your upper chest and the other hand on your diaphragm muscle (i.e., right under your ribs). Notice your hand movements and how fast you are breathing. Deeper, more relaxed breathing will result in your hands being mostly still. Take in normal amounts of air and breathe smoothly.

Now practice slower, deeper breathing and see if your hands start to move less. After you become more practiced at slower, deeper breathing, practice with your hands simply resting at your side.

**Step 2:**

Focus your attention on your breathing. Notice the feeling of air coming in, and after a brief pause, air being exhaled. Notice these feelings and let your attention focus there as best you can.

If you find yourself feeling more relaxed, as you exhale think the word "relax".

*Use your skill when needed:*

If you experience a situation where you feel more anxiety or distress, use this relaxation skill as best you can. Possibly pause or slow down, notice your breathing and slow it down and breathe deeper into you’re your lungs (breathe diaphragmatically). Focus your attention on your breathing for a moment and think “relax” as you feel yourself exhale.
